# Utility of Endoscopic Full-Thickness Resection for Refractory Rectal Bleeding in Chronic Radiation Proctitis

**DOI:** 10.14309/crj.0000000000001556

**Published:** 2024-11-09

**Authors:** Benjamin I. Richter, Yazan Abboud, Ben Flikshteyn, Kaveh Hajifathalian

**Affiliations:** 1 Department of Medicine, Rutgers New Jersey Medical School, Newark, NJ

**Keywords:** radiation proctitis, endoscopic full thickness resection, full thickness resection device, refractory rectal bleeding

## Abstract

Severe rectal bleeding is a rare complication of chronic radiation proctitis (CRP). Given CRP's propensity to involve the full thickness of the rectal tissue, we proposed that endoscopic full-thickness resection may be a successful therapeutic modality for treating CRP. A 76-year-old man with multiple comorbid conditions who was not a surgical candidate presented with severe bleeding secondary to CRP that was refractory to all medical and therapeutic interventions. An endoscopic full-thickness resection was performed, which initially resulted in hemostasis, but the patient ultimately developed recurrent rectal bleeding, and endoscopic resection was determined to be unsuccessful.

## INTRODUCTION

Radiation proctitis (RP) refers to damage to the rectal epithelium caused by secondary ionizing radiation. Depending on the timing of symptom onset, RP is classified as either acute (ARP) or chronic radiation proctitis (CRP).

ARP is a common complication of radiotherapy and is characterized by rectal urgency and pain, diarrhea, and intermittent rectal bleeding.^1^ It involves the superficial mucosa only and is typically self-limiting, usually resolving within 3 months.^2^

CRP is a distinct clinical entity from ARP; it develops at least 6 months after radiation exposure, with reports of symptom onset up to 30 years after radiation.^3^ It involves the full thickness of the rectal tissue, and its occurrence depends on the dose of radiation and volume of irradiated tissue.^4^ Endoscopic findings of CRP are variable, and there are no consensus endoscopic criteria for diagnosis, but mucosal pallor, erythema, loss of vascular pattern, and ulceration are commonly seen.^5^ Severe rectal bleeding is a rare complication of CRP and is difficult to treat because of damage of the underlying mucosa. It frequently recurs and can be refractory to both traditional medical and endoscopic management approaches.

Endoscopic full-thickness resection (EFTR) has emerged as a novel technique for resection of submucosal tumors of the gastrointestinal tract.^6^ Given CRP's propensity to involve the full thickness of the rectal tissue, we proposed that EFTR may be a successful therapeutic modality for treating chronic complications of CRP. A review of the literature revealed no previous reports of EFTR use in the management of severe CRP complications. We report a case of refractory rectal bleeding secondary to CRP and the results of our attempt at using EFTR as a salvage treatment modality.

## CASE REPORT

**Figure 1.**
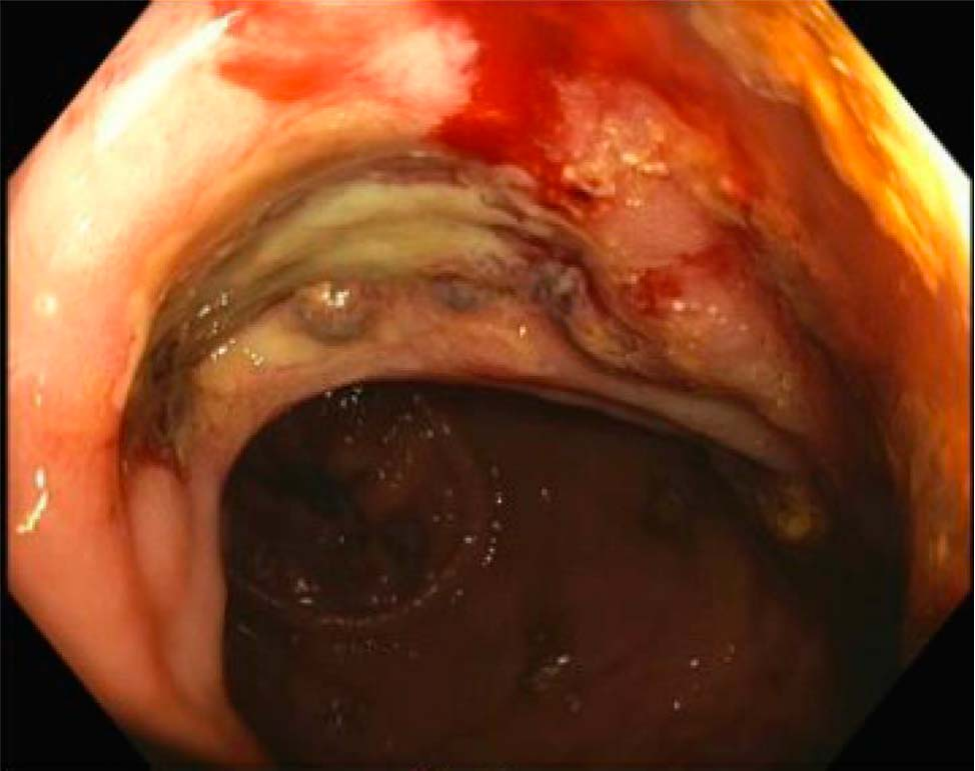
Colonoscopy at time of chronic radiation proctitis diagnosis, which was notable for rectal ulcers with mucosal friability and bleeding.

**Figure 2.**
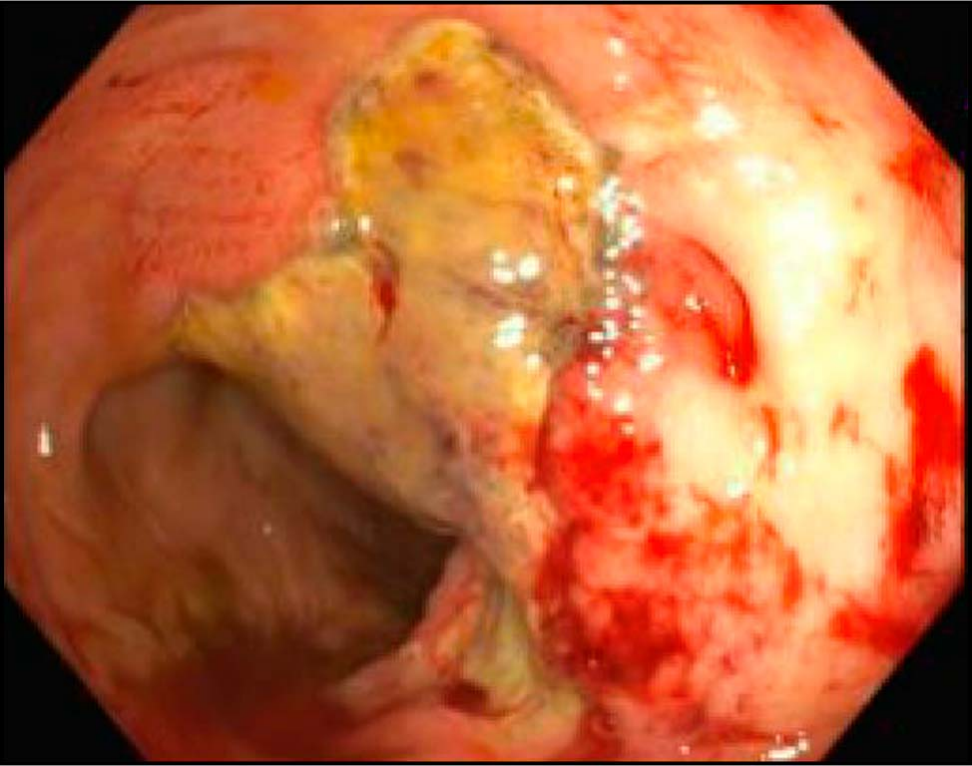
Colonoscopy 1 month after initial chronic radiation proctitis diagnosis, which demonstrated 2 nonbleeding ulcers with ongoing mucosal friability and bleeding.

**Figure 3.**
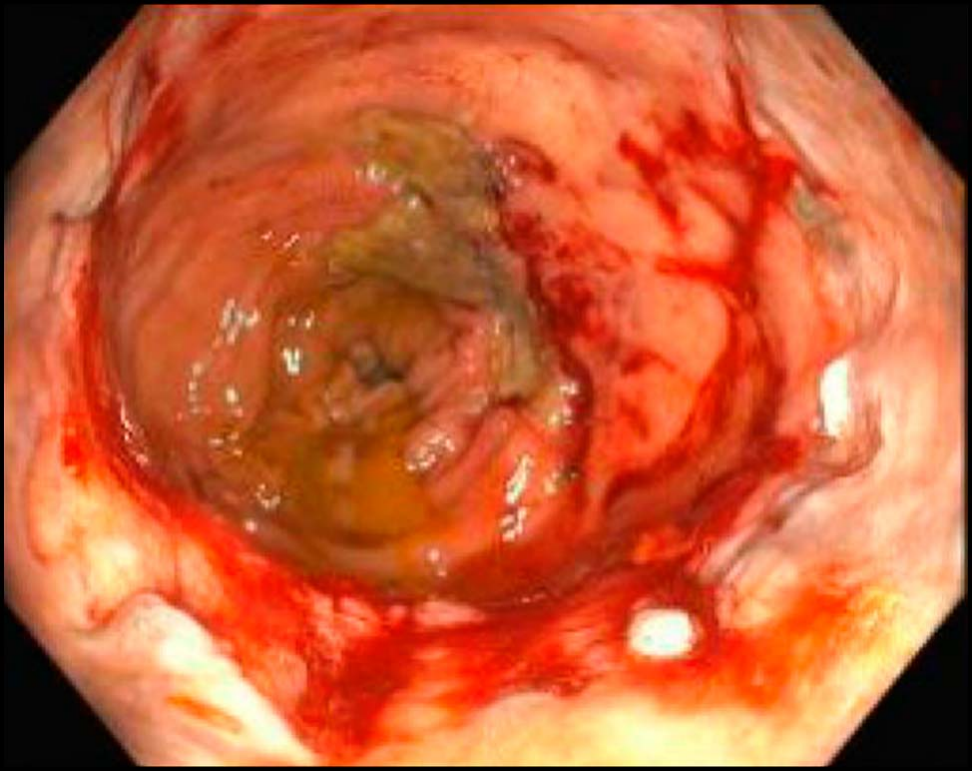
Colonoscopy 1 month after initial chronic radiation proctitis diagnosis, which demonstrated 2 nonbleeding ulcers with ongoing mucosal friability and bleeding.

**Figure 4.**
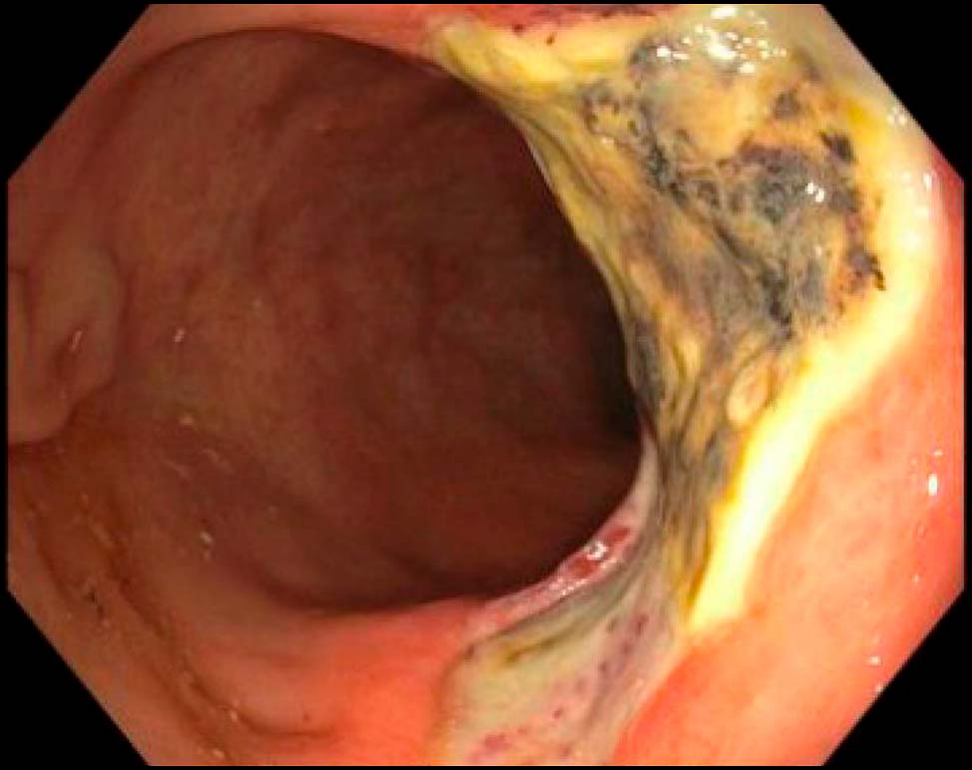
Colonoscopy approximately 2 years after initial diagnosis, which was notable for 2 large (>2-cm) rectal ulcers with friable mucosa, which was treated with APC. Photograph above is after APC treatment. APC, argon plasma coagulation.

**Figure 5.**
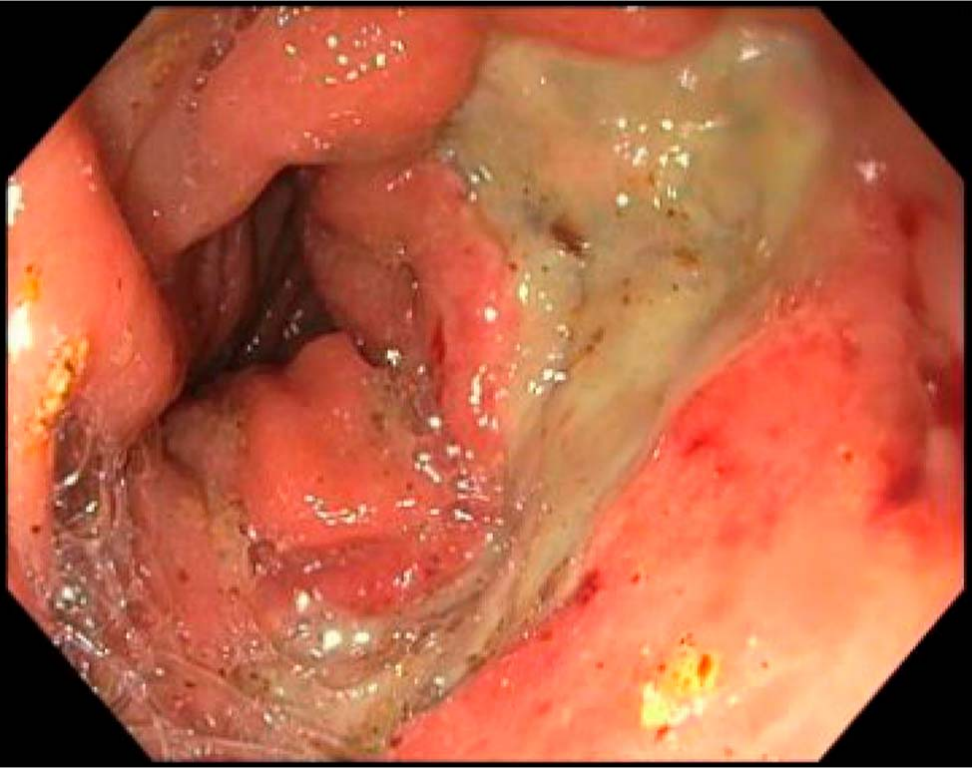
Colonoscopy just before endoscopic full-thickness resection.

**Figure 6.**
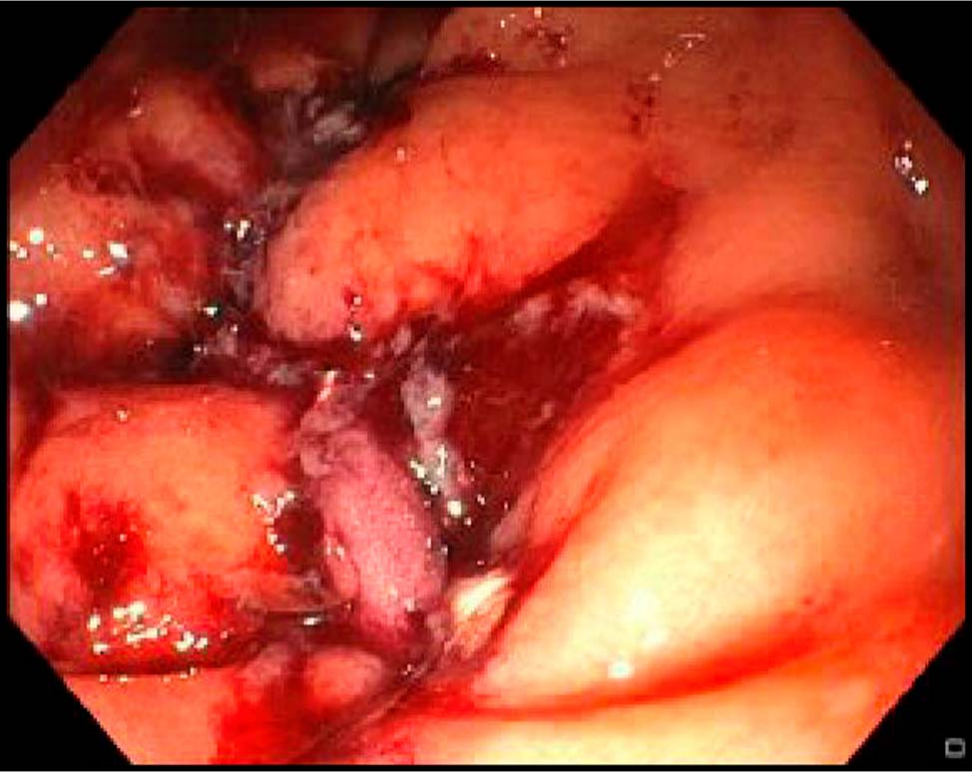
Colonoscopy on completion of endoscopic full-thickness resection.

**Figure 7.**
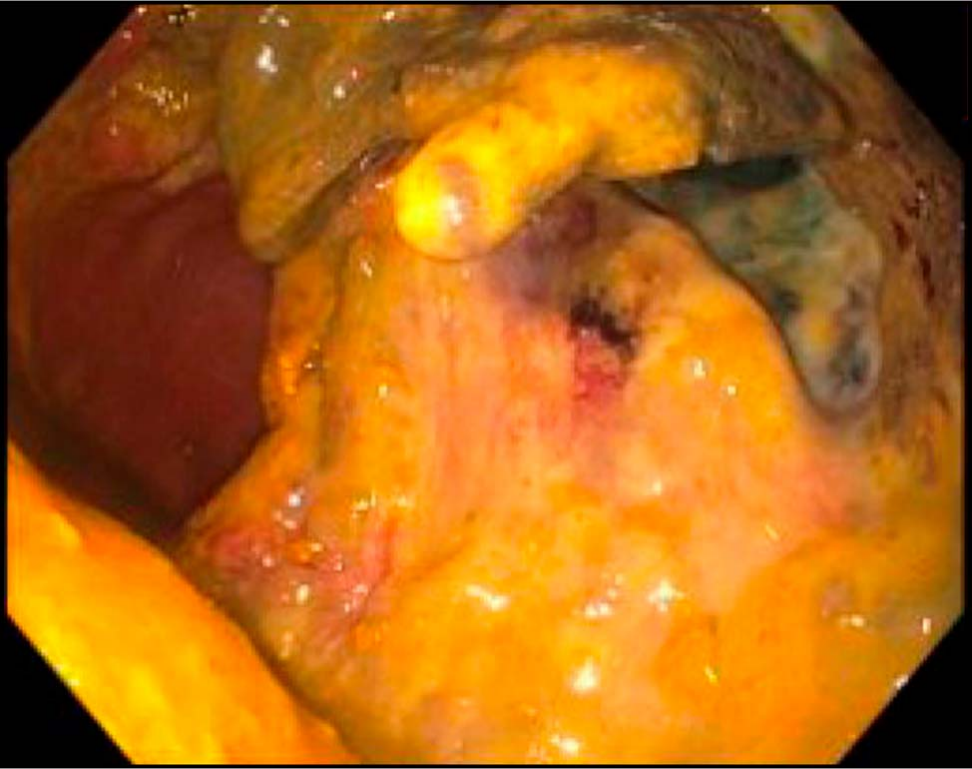
Flexible sigmoidoscopy 4 months after completion of endoscopic full-thickness resection, which revealed a large ulcer in the rectum with old clots attached without fistula formation.

A 76-year-old man with medical history of end-stage renal disease on hemodialysis, coronary artery disease, heart failure, atrial flutter on apixaban, chronic constipation, and rectal prolapse underwent radiation therapy for treatment of prostate cancer. Six months after radiation, he developed rectal bleeding. Colonoscopy at the time was notable for rectal ulcers with mucosal friability and bleeding (Figure 1), for which a hemostatic clip was placed. Given his constellation of symptoms and endoscopic findings, he was diagnosed with CRP. In the following month, he continued to have intermittent hematochezia, for which a repeat colonoscopy was performed, which demonstrated 2 nonbleeding ulcers with ongoing mucosal friability and bleeding that subsided without intervention (Figures 2 and 3). There were no subsequent episodes of bleeding over the following few months. He then underwent placement of a drug-eluting stent for severe coronary artery disease; he started taking clopidogrel and continued taking apixaban. A few weeks after initiating clopidogrel, he developed recurrent rectal bleeding, with colonoscopy showing rectal angioectasias and rectal ulcers; the angioectasias were treated with argon plasma coagulation (APC). He completed a hydrocortisone suppository course for 4 weeks, too. Two years later, his bleeding recurred and was complicated by severe anemia and hemodynamic instability. He underwent multiple embolizations of the hemorrhoidal artery by interventional radiology (IR). He then underwent a colonoscopy, which was notable for 2 large (>2-cm) rectal ulcers with friable mucosa, which were treated with APC (Figure 4, post-treatment); the ulcers were too large for placement of a hemostatic clip. He experienced ongoing rectal bleeding, so he underwent endoscopic suturing (X-Tack; Apollo Endosurgery, Austin, TX) and IR–guided embolization of the superior rectal artery. After both attempts at hemostasis were unsuccessful, the case was discussed with the surgical service, but the patient was deemed not a surgical candidate, so the decision was made to perform a free-hand EFTR of a large bleeding ulcer. This procedure was conducted as a salvage intervention after multidisciplinary discussion of the case. Rectal ulcer biopsy at the time was notable for colonic mucosa with ulceration, acute inflammation, and granulation tissue formation (Figure 5). Full-thickness resection of the ulcer was performed—using a gastroscope with a transparent cap and various endoscopic knives—down to the perirectal fascia. A double-channel endoscope with an affixed endoscopic suturing system (OverStitch; Apollo Endosurgery) was used to place 5 running sutures over the defect with adequate tissue approximation (Figure 6), and no bleeding was noted at the end of the procedure. Repeat colonoscopy 3 days later revealed intact endoscopic closure without active bleeding or fistula formation. At the time of discharge, blood counts were stable, and he had no ongoing rectal bleeding. On follow-up 30 days after EFTR, the patient had no ongoing rectal bleeding with stable chronic anemia. Four months later, he presented with recurrent hematochezia. Flexible sigmoidoscopy revealed a large ulcer in the rectum with old clots attached without fistula formation, proving previous endoscopic resection to be unsuccessful (Figure 7). The patient died of a cardiac event 4 days after endoscopy, while still hospitalized.

## DISCUSSION

Refractory bleeding from CRP is a challenging complication to treat because of the underlying vulnerability of the irritated rectal tissue. Although both endoscopic and medical management techniques have been proposed in the literature, there remains a considerable rate of recurrence with the standard approaches.^7,8^ In the current case, the patient failed conservative medical management with laxatives and topical steroids; he also failed multiple endoscopic and interventional radiology treatment modalities, including hemostatic clip placement, APC, endoscopic suturing, and multiple embolizations.

EFTR has emerged as a novel technique for resecting submucosal tumors that involves deep muscular tissue; however, there is minimal literature on its utility for hemostasis in refractory bleeding and no previous reports of its use in managing bleeding from CRP. Our team trialed this approach because of several factors: First, CRP tends to involve the full thickness of the rectal tissue; second, the patient's underlying risk factors—such as chronic constipation, intermittent rectal prolapse, end-stage renal disease on dialysis, and antiplatelet therapy—would continue to aggravate the underlying damage and prevent healing through other treatment modalities; and third, the failure of all other attempts at medical or endoscopic management in a nonsurgical patient.

Despite no recurrence of hematochezia in the 30-day post-op course, our patient had bleeding recurrence after a few months, with evidence of incomplete healing of the resected portion of the rectum. Although it is unknown to what extent the patient's underlying risk factors for aggravation of the rectal tissue and bleeding contributed to his outcome, given our experience, we recommend against the use of EFTR for the treatment of refractory bleeding in patients with CRP.

## DISCLOSURES

Author contributions: BI Richter: substantial contributions to the conception and design of the work; the acquisition, analysis, and interpretation of data for the work; and the drafting of the work. Y. Abboud: drafting parts of the work; revising the work critical for important intellectual content. BD Flikshteyn: drafting parts of the work; revising the work critical for important intellectual content. K. Hajifathalian: revising the work critical for important intellectual content, and is the guarantor of this article.

Financial disclosure: None to report.

Informed consent was obtained for this case report.
